# Insight into the functional roles of Glu175 in the hyperthermostable xylanase XYL10C-ΔN through structural analysis and site-saturation mutagenesis

**DOI:** 10.1186/s13068-018-1150-8

**Published:** 2018-06-08

**Authors:** Shuai You, Chun-Chi Chen, Tao Tu, Xiaoyu Wang, Rui Ma, Hui-yi Cai, Rey-Ting Guo, Hui-ying Luo, Bin Yao

**Affiliations:** 1 Key Laboratory for Feed Biotechnology of the Ministry of Agriculture, Feed Research Institute, Chinese Academy of Agricultural Sciences, Beijing, 100081 China; 2 National Engineering Laboratory of Industrial Enzymes, Tianjin Institute of Industrial Biotechnology, Chinese Academy of Sciences, Tianjin, 300308 China; 3 College of Life Sciences, Hubei University, Wuhan, 430062 China

**Keywords:** GH10 xylanase, Catalytic efficiency, Site-saturation mutagenesis, Biofuel industry

## Abstract

**Background:**

Improving the hydrolytic performance of hemicellulases to degrade lignocellulosic biomass is of considerable importance for second-generation biorefinery. Xylanase, as the crucial hemicellulase, must be thermostable and have high activity for its potential use in the bioethanol industry. To obtain excellent xylanase candidates, it is necessary to understand the structure–function relationships to provide a meaningful reference to improve the enzyme properties. This study aimed to investigate the catalytic mechanism of a highly active hyperthermophilic xylanase variant, XYL10C-ΔN, for hemicellulose degradation.

**Results:**

By removing the N-terminal 66 amino acids, the variant XYL10C-ΔN showed a 1.8-fold improvement in catalytic efficiency and could hydrolyze corn stover more efficiently in hydrolysis of corn stover; however, it retained similar thermostability to the wild-type XYL10C. Based on the crystal structures of XYL10C-ΔN and its complex with xylobiose, Glu175 located on loop 3 was found to be specific to GH10 xylanases and probably accounts for the excellent enzyme properties by interacting with Lys135 and Met137 on loop 2. Site-saturation mutagenesis confirmed that XYL10C-ΔN with glutamate acid at position 175 had the highest catalytic efficiency, specific activity, and the broadest pH-activity profile. The functional roles of Glu175 were also verified in the mutants of another two GH10 xylanases, XylE and XynE2, which showed increased catalytic efficiencies and wider pH-activity profiles.

**Conclusions:**

XYL10C-ΔN, with excellent thermostability, high catalytic efficiency, and great lignocellulose-degrading capability, is a valuable candidate xylanase for the biofuel industry. The mechanism underlying improved activity of XYN10C-ΔN was thus investigated through structural analysis and functional verification, and Glu175 was identified to play the key role in the improved catalytic efficiency. This study revealed the importance of a key residue (Glu175) in XYN10C-ΔN and provides a reference to modify GH10 xylanases for improved catalytic performance.

**Electronic supplementary material:**

The online version of this article (10.1186/s13068-018-1150-8) contains supplementary material, which is available to authorized users.

## Background

The rising global demand for renewable fuels and the climate change associated with the consumption of fossil fuels are considered as the biggest social challenges over the next few decades [1, 2]. Bioethanol produced from polysaccharides such as starch can substitute for fossil fuel and has been identified as the first-generation biofuel; however, its use so far has been restricted as it is not sustainable. For second-generation biofuels, renewable resources including wood, grass, agricultural and forest residues, and fiber sludge are widely used for bioethanol production [3–5]. Biomass mainly comprises cellulose, hemicellulose and lignin, in which the cellulose fibers are embedded in a matrix of hemicellulose and lignin. Therefore, to convert biomass into fermentable sugars efficiently, the synergistic actions of hemicellulases and cellulases are very important [6–8].

Hemicellulose is the second most abundant polysaccharide on earth. Efficient conversion of hemicellulose to other value-added fermentation products has both environmental and economic benefits [9]. Xylan, the major component of hemicellulose, consists of a backbone of xylose units, linked via β-1,4-glycosidic bonds and some branched chains. Owing to the structural complexity, complete degradation of xylan requires a series of enzymes. Among them, xylanase (endo-1,4-d-xylanase, EC 3.2.1.8) is a key enzyme that randomly cleaves the internal β-1,4-d-xylosidic linkages of xylan to yield different chain lengths of xylo-oligosaccharides [10]. Based on their amino acid sequence similarities, three-dimensional (3-D) structures, and hydrophobic cluster analysis, xylanases are usually classified into glycoside hydrolase (GH) families 10 and 11 [9]. GH10 xylanases have a typical (β/α)_8_ barrel fold structure (also known as the typical triosephosphate isomerase barrel) with catalytic residues at the end of β-sheets 4 and 7 [10, 11], while GH11 xylanases display a β-jelly roll structure and employ the same retaining mechanism as GH10 xylanases [12, 13].

Currently, the commercial uses of xylanases are mainly in the paper, food, and animal feed industries [9, 14], however, xylanases are increasingly being recognized as important enzymes in biorefining of lignocellulosic biomass [15, 16]. In the production of bioethanol from lignocellulosic materials, xylanase can promote the hydrolysis of cellulose by degrading xylan and increasing the access of cellulase to the cellulose surface [17]. GH10 xylanases have a broad substrate specificity and, in combination with cellulases, degrade wheat straw and corn fiber very efficiently [18–20]. The poor catalytic performance of most wild-type xylanases restricts their application in bioprocesses [21]; therefore, it is desirable to determine the catalytic mechanism of xylanase and improve its properties by protein engineering. Some structural elements, such as local loops and terminal regions, and key residues involved in the formation of protein–protein interactions (e.g., hydrogen bonds, salt bridges, and sigma *π*), fold on triggered trimerization, which affects the catalytic process of proteins, making them potential foci for protein engineering [22–27]. Among these factors, some key residues in the active loops and the N-terminus play certain roles in the catalytic efficiency of xylanases [25, 26]. Their functions have been verified by site-directed and saturation mutagenesis, based on the simple but powerful Darwinian evolution principles of mutation and selection [28, 29].

To meet the rigorous requirements of industrial processes, and to decrease the production-cost, highly active xylanases with excellent thermostability are of great interest. The GH10 xylanase, XYL10C, from acidophilic *Bispora* sp. MEY-1 is an attractive candidate industrial enzyme that has remarkable activity and favorable thermostability [30]. The present study aimed to construct a truncated mutant, XYL10C-ΔN, and compare its enzymatic properties with those of the wild-type to reveal the underlying mechanisms of its high catalytic efficiency, and to form the basis for improving other GH10 xylanases.

## Results

### Characterization of the N-terminus-truncated mutant XYL10C-ΔN

Based on the multiple sequence alignment of GH10 xylanases (Additional file 1: Figure S1), XYL10C was observed to harbor an extra N-terminal sequence of 66 amino acid residues (except for the 18-residue signal peptide). The mutant version of XYL10C with the N-terminal sequence deleted, XYL10C-ΔN, was expressed in *Pichia pastoris* and purified (Additional file 1: Figure S2). In comparison with the wild-type XYL10C (optimally active at 85 °C and pH 4.5), XYL10C-ΔN showed a slight downshift of 5 °C and 0.5 in the optimal temperature and pH (Table 1). The two enzymes had similar kinetic stabilities at 80 °C and 90 °C, but different thermodynamic stabilities (Additional file 1: Figure S3). Compared with XYL10C, XYL10C-ΔN had a decreased *T*_m_ value of 73.8 °C (vs. 82.0 °C). The enzymes had similar pH stability (Additional file 1: Figure S4) and employed an endo-mode of action to cleave xylotriose, xylotetraose, xylopentaose, xylohexaose, and beechwood xylan into mainly xylose and xylobiose (Additional file 1: Figure S5). Significant differences were observed for their substrate affinities and catalytic performances. XYL10C-ΔN had higher *K*_m_, *k*_cat_/*K*_m_ (catalytic efficiency), and specific activity values (Table 1), which were 1.3-, 2.7-, and 1.8-fold higher than those of XYL10C, respectively.

**Table 1:** Enzymatic properties and kinetic values of purified XYL10C, XYL10C-ΔN, XylE, XynE2, and their mutants with beechwood xylan as the substrate

Enzymes	Optimal pH	Optimal temperature (°C)	Specific activity (U/mg)	*K*_m_ (mg/mL)	*k*_cat_/*K*_m_ (mL/s/mg)
XYL10C	4.5	85	3200 ± 131	0.54 ± 0.02	4900 ± 201
XYL10C-ΔN	4.0	80	8700 ± 403	0.71 ± 0.02	8800 ± 403
XYN10C-ΔN-E175Q	5.0	85	3600 ± 167	0.73 ± 0.02	4400 ± 198
XylE	5.0	70	620 ± 28	1.01 ± 0.03	490 ± 20
XylE-Q116E	5.5	70	2300 ± 108	0.81 ± 0.02	1200 ± 38
XynE2	8.0	65	870 ± 37	0.93 ± 0.03	1600 ± 39
XynE2-Q85E	7.0	65	1100 ± 49	0.72 ± 0.02	2200 ± 91

Values represent mean ± SD (*n* = 3)

### Analysis of the crystal structures of XYL10C-ΔN and its complex

The crystal structure of XYL10C-ΔN was solved at a high resolution of 1.6 Å by molecular replacement using the structure of XylE (PDB code: 4F8X) from *Penicillium scopiformis* [31] as a search model. The structure determination procedures are listed in Additional file 1: Table S1. Based on the analysis of the X-ray diffraction pattern (Additional file 1: Figure S6), the XYL10C-ΔN crystals belonged to the monoclinic space group of C2. As shown in Additional file 1: Figure S7, there are two monomers, which have the topology of a single (β/α)_8_ barrel typical of GH10 xylanases, and are denoted as chain A and B in the asymmetric unit. Strong protein interactions were detected between chain A and B, including hydrogen bonds and van der Waals forces. These tightly fixed subunits probably have a better ability to bind the substrate at the catalytic pocket and maintain the stability of the monomer.

**Fig. 1:**
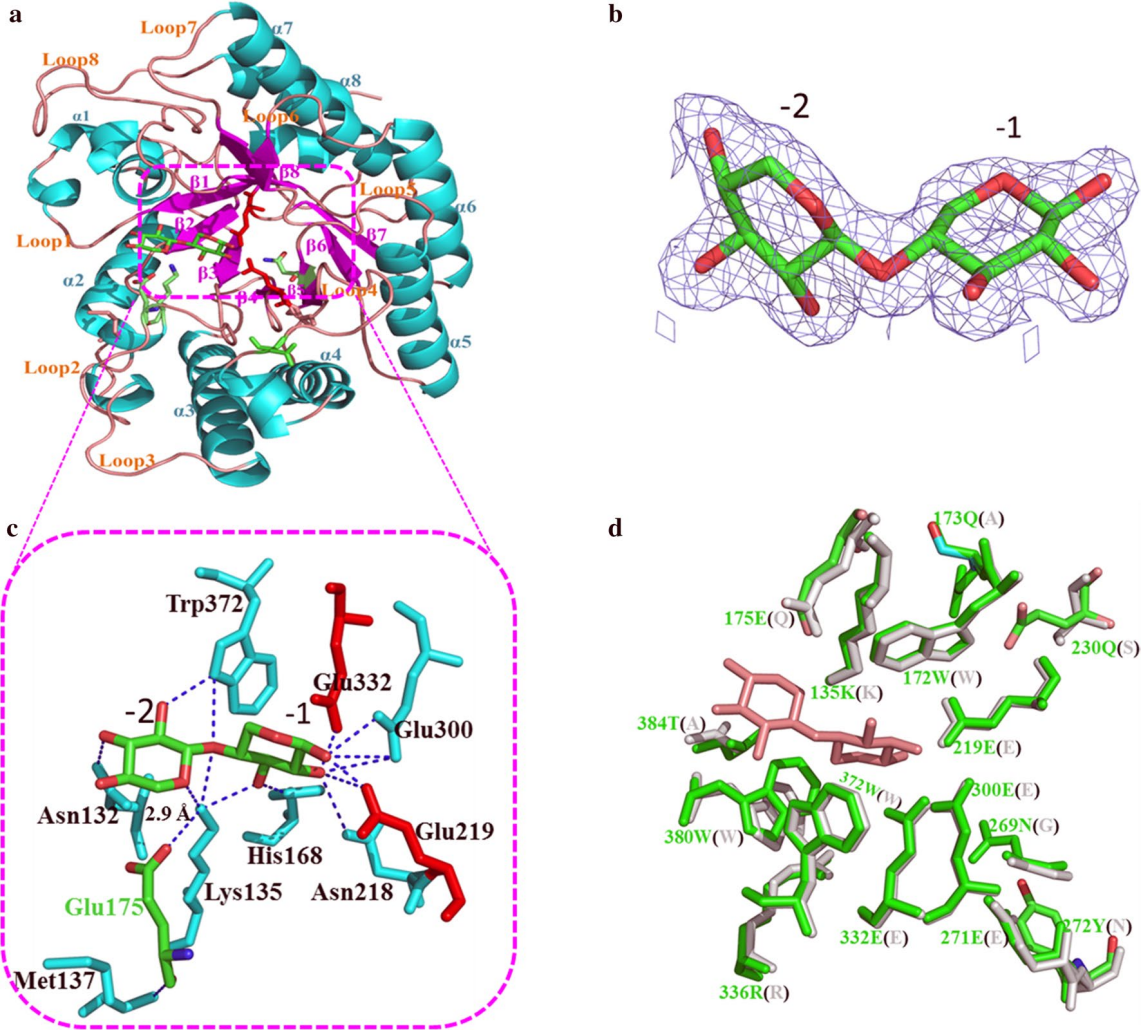
The overall structure and active-site architecture of the XYL10C-ΔN-xylobiose complex. **a** The crystal structure of XYL10C-xylobiose complex. The typical (β/α)_8_ barrel fold of XYL10C-ΔN with α-helices (cyan), β-strands (violet) and loops (pink) indicated. The loops at the C-terminus of the β-strands form a salad bowl-shaped groove that accounts for the endo-mode action against polymeric substrates. The catalytic residues Glu219 and Glu332, located on strands β4 and β7, are involved in substrate recognition and catalysis. **b** The electron density map. **c** Xylobiose (green) binding in the active site of XYL10C-ΔN. Catalytic residues are indicated in red. Amino acids that are important for to bind xylobiose residues in the subsites are indicated. **d** Structural comparison of XYL10C-ΔN and XylE (PDB: 4F8X; green). The amino acids of XylE are shown in brackets

To fully understand the substrate binding and catalysis mechanisms, co-crystallization of XYL10C-ΔN and xylobiose was also completed at a resolution of 1.7 Å. As shown in Fig. 1, there is one xylobiose molecule in the open and extended active-site groove of XYL10C-ΔN, and two β-1,4-linked xylosyl moieties in subsites − 1 and − 2. Superimposition of the XYL10C-ΔN-xylobiose complex structure and the XYL10C-ΔN structure yielded a root-mean-square deviation (RMSD) value of 0.24 Å for all Cα atoms, suggesting that ligand binding did not alter the protein conformation. The xylobiose can form several hydrogen bonds, such as with Ala131, Asn132, His168, Asn218, Glu300, and Trp372 around the catalytic pocket, which provide strong binding forces to stabilize the enzyme–substrate complex and to maintain effective catalysis. Among them, Ala131, Lys135, and Met138 are located on loop 2, which is the active loop involved in substrate binding and catalysis. Thus, the amino acid residues that can affect the swing of loop 2 will affect the catalytic efficiency of the enzyme. By docking with long-chain xyloheptaose (Additional file 1: Figure S8), Asn269, Tyr272, His302, and Arg336 were also found to interact with the substrate. These residues are conserved throughout evolution and indispensable for substrate binding and catalysis.

### Identification of the key residue

As shown in the structure of the XYL10C-ΔN-xylobiose complex (Fig. 1c), the side chain N atom of Lys135, located on the loop 2, forms three hydrogen bonds with the hydroxyl oxygen, glycosidic oxygen, and O5 of xylobiose. These interactions limit the swing of loop 2, and further affect the interactions of the substrate and other functional amino acids. The OE2 of Glu175 shows a distance of approximately 2.9 Å to the side chain N atom of Lys135, which permits the formation of salt and hydrogen bonds. Moreover, the carboxy oxygen of Glu175 can form a hydrogen bond with the nitrogen of Met137 located on loop 2. In addition, loop 3, located in the catalytic pocket, would be immobilized because of the counteracting force. The strictly conserved Trp172 in loop 3 can form a hydrogen bond with the catalytic Glu219 and exerts a hydrophobic force with xylopyranose. Glu175 is located in the center of this powerful interaction network and contributes to the stable conformation of loop 3, the loop responsible for the rapid catalytic reaction. Multiple sequence alignment of 50 GH10 xylanases indicated that four proteins have Glu at this site (Table 2). Thus, residue Glu175 of XYL10C-ΔN, which interacts strongly to Lys135 and Met137 of loop 2, and is in close proximity to loop 3, might have the roles of stabilizing the substrate conformation in the catalytic center and affecting the catalytic efficiency of XYL10C-ΔN. To verify the functional roles of Glu175 in the catalysis of GH10 xylanases, site-saturation mutagenesis was performed.

**Table 2:** Conserved degrees of the amino acid residues located in the active pockets and catalytic channels of XYL10C-ΔN and other 49 GH10 counterparts

Amino acids^a^	Location	Conserved degree^b^	Statistics
272 Tyr (Asn)	Active pocket	9	15× Y, 10× G, 6× S, 3× D, 3× R, 3× T, 3× W, 2× N, 2× Q, 1× A,1× E,1× F
307 Glu (Glu)	Active pocket	7	15× Q, 7× E, 7× S, 5× G, 2× A, 2× N, 2× R, 2× V, 1× D, 1× H, 1× L, 1× M, 1× P, 1× T
384 Thr (Ala)	Active pocket and catalytic channel	5	25× T, 14× V, 3× S, 2× F, 1× A, 1× E, 1× Q, 1× W
173 Gln (Ala)	Active pocket	2	35× H, 11× Y, 2× Q, 1× A, 1× D
269 Asn (Gly)	Active pocket	2	42× N, 3× G, 3× S, 1× A
230 Gln (Ser)	Active pocket	1	44× R, 2× Q, 1× K, 1× S, 1× V
175 Glu (Gln)	Active pocket and catalytic channel	1	44× Q, 4× E, 1× G, 1× P
300 Glu (Glu)	Active pocket	1	42× Q, 8× E
135 Lys (Lys)	Active pocket and catalytic channel	1	49× K, 1× D
168 His (His)	Active pocket and catalytic channel	1	49× H, 1× D
372 Trp (Trp)	Active pocket and catalytic channel	1	50× W
218 Asn (Asn)	Active pocket	1	49× N

^a^Position and amino acid of XYL10C-ΔN (the corresponding residue in XylE)

^b^The lower values correspond to higher conservation

### Characterization of XYL10C-ΔN mutants

**Fig. 2:**
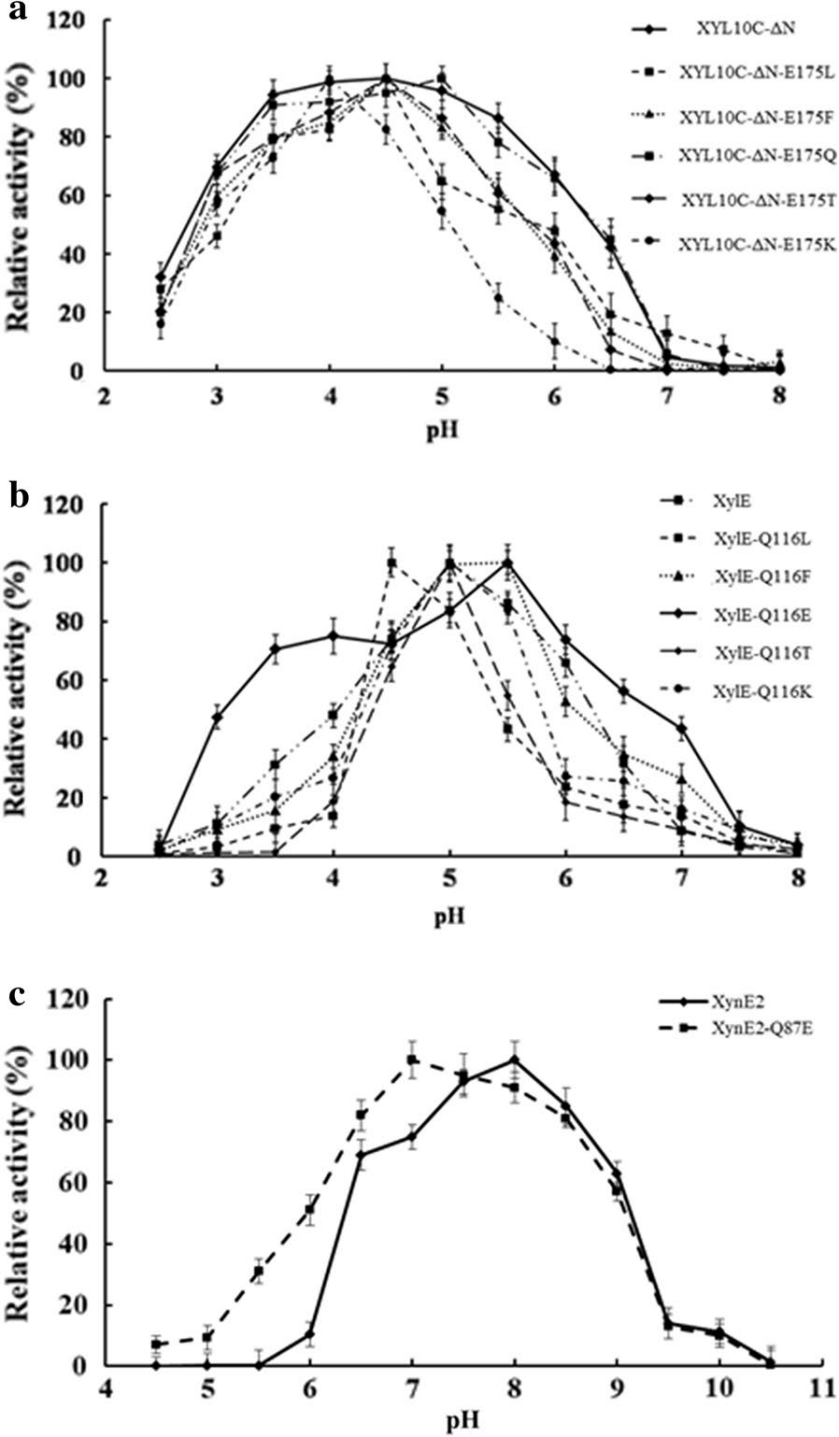
The pH-activity profiles of XYL10C-ΔN, XylE, XynE2, and their representative mutants. **a** XYL10C-ΔN and its mutants; **b** XylE and its mutants; **c** XynE2 and its mutant

**Fig. 3:**
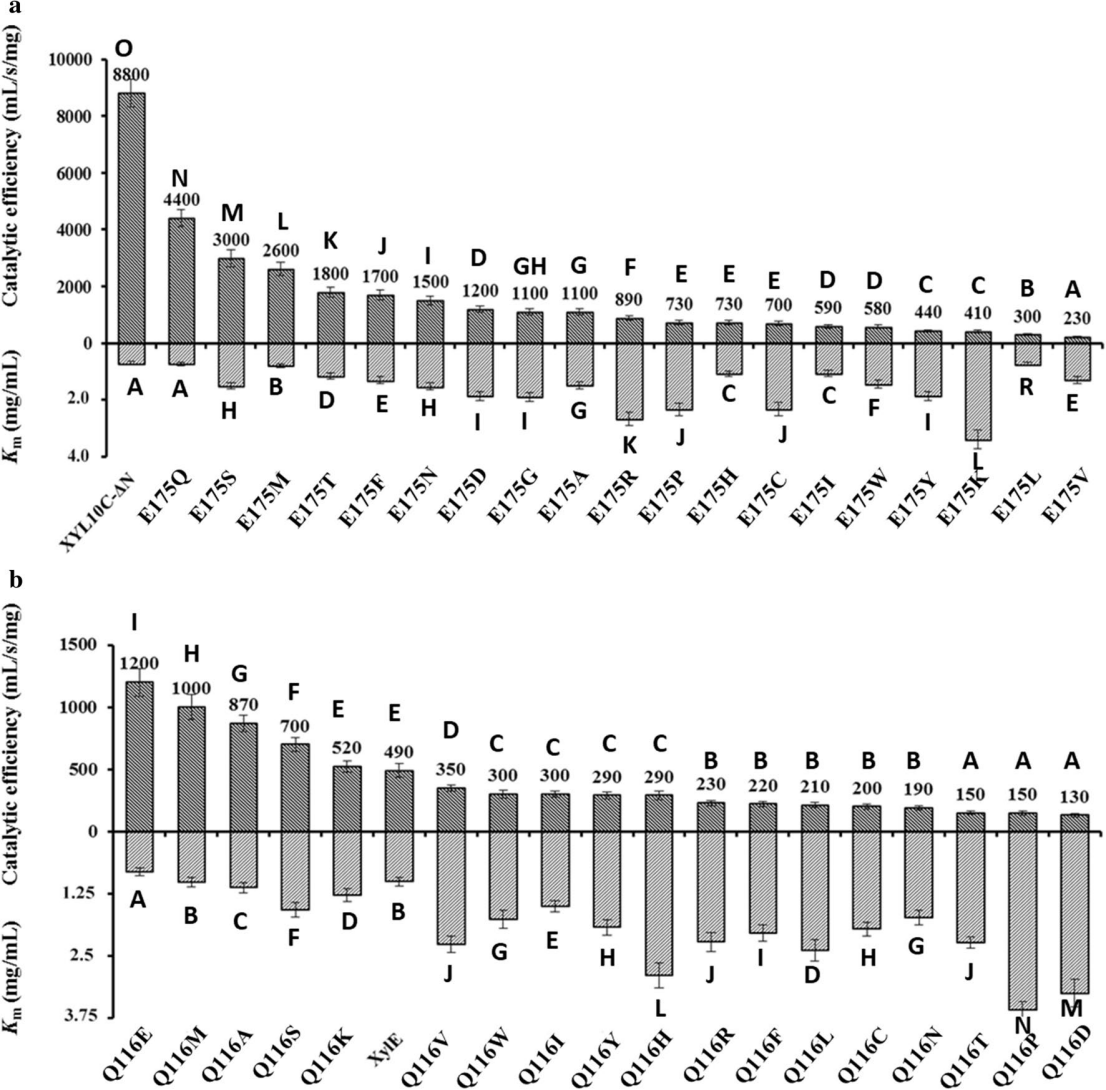
The catalytic efficiencies (*k*_cat_/*K*_m_) and *K*_m_ values of XYL10C-ΔN, XylE, and their mutants. **a** XYL10C-ΔN and its mutants; **b** XylE and its mutants. Different letters mean significant difference at *P* < 0.05

XYL10C-ΔN mutants with Glu175 replacement were successfully constructed, expressed, and purified (Additional file 1: Figure S2). Compared with XYL10C-ΔN, the variant enzymes showed a temperature optimum change of − 5 to + 15 °C and a pH optimum change of − 0.5 to + 1.0 (Additional file 1: Table S2). Moreover, the mutants had narrower pH-activity profiles (Fig. 2a). Using beechwood xylan as the substrate, the mutants had increased *K*_m_ values (1.1–4.8-fold) and decreased *k*_cat_/*K*_m_ values (1.0–37.3 times), as well as decreased specific activities (0.8–32.5 times) (Fig. 3a and Additional file 1: Table S3). The catalytic efficiency of XYL10C-ΔN was significantly different from that of all other site-mutated variants (*P* < 0.05). The *K*_m_ values of XYL10C-ΔN and XYL10C-ΔN-E175N were very similar, but were significantly different from those of other variants. The results indicated that residue substitution at position 175 probably altered the intramolecular interaction, the protein structure, or the substrate conformation, which consequently changed the enzyme’s properties.

### Functional validation of Glu175 in other GH10 xylanases

Two xylanases, XylE and XynE2, were selected to verify the roles of Glu175 in the catalytic performance and pH adaptability of GH10 xylanases. Compared with the wild-type proteins, residue substitution caused no changes in the temperature and pH optima of most mutants (65–70 °C and 5.0–5.5; Additional file 1: Table S4); however, mutants XylE-Q116E and XynE2-Q85E showed broader pH-activity profiles (Fig. 2b, c), increased catalytic efficiencies (by 1.2 and 9.2-fold, respectively), and specific activities (by 1.1 and 7.7-fold, respectively) (Table 1, Additional file 1: Table S5 and Fig. 3b). The catalytic efficiency and *K*_m_ value of XylE-Q16E were different from those of all other variants (*P* < 0.05). These results confirmed the functional roles of Glu175 in the catalytic performance and pH-activity profile of GH10 xylanases. However, the importance of Glu175 in the catalytic efficiency of other GH10 xylanases remains to be determined.

### Enzymatic hydrolysis of corn stover

**Fig. 4:**
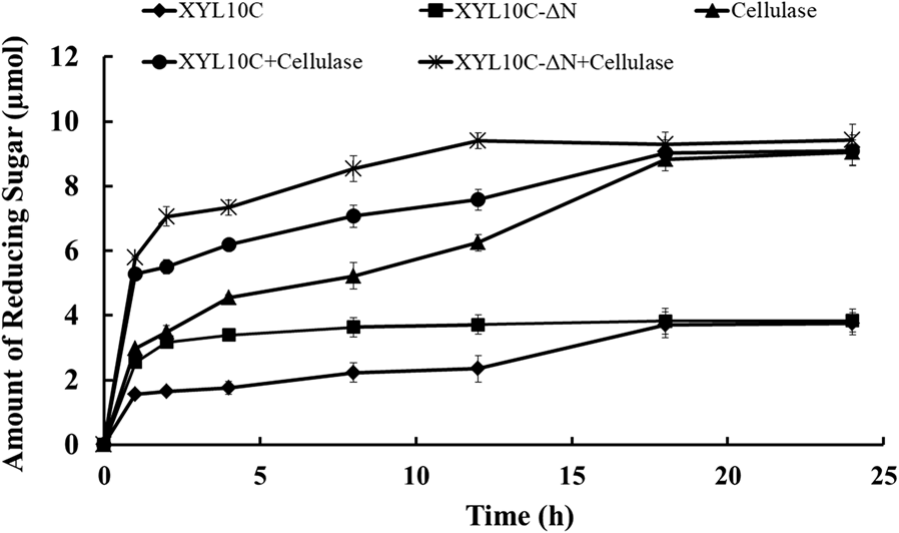
The hydrolysis courses of pretreated corn stover by *T. reesei* cellulase (100 U) and its combinations with XYL10C and XYL10C-ΔN (30 U xylanase and 70 U cellulase) at 50 °C and pH 5.0

Steam-exploded corn stover mainly comprises cellulose and hemicellulose. When using steam-exploded corn stover as the substrate, the hydrolytic capabilities of commercial cellulase from *T. reesei* and its combination with XYL10C or XYL10C-ΔN were assessed at pH 5.0 and 50 °C with agitation for various times. As shown in Fig. 4, 100 U of *T. reesei* cellulase alone released 8.0 µmol of reducing sugars during 24 h of incubation. When combined with XYL10C or XYL10C-ΔN (30 U of xylanase and 70 U of cellulase), the reducing sugar amounts released were 9.1 and 9.4 µmol, and during the first 2 h of reaction, the sugar production rates increased by 2 and 1.6 times, respectively. The results indicated that *T. reesei* cellulase and XYL10C or XYL10C-ΔN have a synergistic activity to degrade steam pretreated corn stover, and that XYL10C-ΔN is more efficient than the wild-type for the hydrolysis of corn stover.

## Discussion

The study of the relationship between enzyme structure and function provides an important reference for efficient protein engineering [32]. Artificial enzyme engineering is a powerful strategy to pinpoint functional determinants and to rapidly improve enzyme fitness with regard to its physical or biochemical properties [33–36]. Thermostable xylanases with high catalytic efficiency are highly desirable for many industrial processes. In our previous study, a hyperthermophilic xylanase of GH10, XYL10C, from *Bispora* sp. MEY-1 showed great application potential in the bioethanol industry [30]. Multiple sequence alignment indicated that XYL10C contains an extra N-terminal sequence of 66 amino acid residues. N-terminal motifs play an important role in protein function, such as binding, thermostability, and specific activity [37–39]. In the present study, after removal of this N-terminal sequence, the variant XYL10C-ΔN retained its hyperthermophilic characteristic but showed a 1.8- and 2.7-fold improvement in catalytic efficiency and specific activity, respectively. XYL10C-ΔN shares high-sequence identity with the *Talaromyces leycettanus* xylanase *Tl*Xyn10A (54%) [40] and *Penicillium canescens* xylanase XylE (53%) [31], but has a higher specific activity (8700 vs. 2240 and 50 U/mg) and catalytic efficiency (8800 vs. 1626 and 92 mL/s/mg). Moreover, XYL10C-ΔN exhibits similar thermostability to *Tl*Xyn10A (no enzyme activity loss after 1 h-incubation at 80 °C), which is better than XylE (completely inactive after 30 min incubation at 70 °C). Compared with other GH10 xylanases, such as *reAu*Xyn10A from *Aspergillus usamii* (5448 mg/mL) [41], the H179F mutant of XynA (1030 mL/s/mg) from *Geobacillus stearothermophilus* [42], *Mp*Xyn10A from *Malbranchea pulchella* [43], and *Gt*Xyn10 from *Gloeophyllum trabeum* [44], XYL10C-ΔN also has a superior catalytic performance and thermostability. Therefore, it is important to reveal the underlying mechanism of highly active XYL10C-ΔN for industrial purposes.

GH10 xylanases have conserved amino acids located in the shallow slot around the top face of the protein molecule, including two catalytic glutamates and five to seven substrate binding subsites [45–48]. Among them, the highly conserved subsites − 1, − 2, and + 1 [48] have several diverse interactions with xylose moieties, and thus play a critical role in substrate recognition and binding. Structural analysis of the XYL10C-ΔN-xylobiose complex indicated five key residues (Asn132, Lys135, His168, Asn218, and Trp372) that are involved in substrate binding via hydrogen bonds. However, ligand binding in the aglycon region of the substrate binding cleft is determined by the hydrophobic interactions between aromatic amino acids and xylobiose rings. Despite the conserved sequence and structure near the glycon subsites, the proximal glycon subsites may display variations. For example, *Cellvibrio japonicus* xylanase 10C, having an insertion of Tyr and a substitution of Glu/Gly at subsites − 3 and − 2, showed very low activity on less polymerized substrates [46]. XYL10C-ΔN and XylE, with 53% sequence similarity, share similar three-dimensional structures; however, their enzyme properties differ considerably [30, 31]. As shown in the structural alignment of XYL10C-ΔN and XylE (Fig. 1d), there are several important but different residues in the catalytic pockets. Among them, Glu175 of XYL10C-ΔN on loop 3, which is in close proximity to many important substrate binding sites and the substrate, might play a part in the formation of the catalytic pocket. In particular, it can form a variety of force interactions with Lys135 on loop 2, the loop that interacts with the substrate to stabilize the conformation. The loop structures, especially the flexible ones, are involved in substrate binding, enzyme catalysis, and product release of triose-phosphate isomerase (TIM)-(β/α)_8_ enzymes [49, 50]. The residue at position 175 is highly conserved in evolution. Most known GH10 xylanases have glutamine at this site, while XYL10C-ΔN has glutamate instead. Site-saturation mutagenesis of XYL10C-ΔN indicated that this site is closely associated with the catalytic efficiency, and Glu, Met, Ser, and Gln were the dominant residues for the catalytic efficiency and specific activity.

Xylanases with high specific activity, good catalytic efficiency, and excellent stability are required for biorefinery uses. Recent studies have shown that the longer the xylo-oligosaccharides, the greater the extent of cellulase inhibition [51]. In addition, cellulases stuck on cellulose microfibrils during enzymatic hydrolysis show a decreased degradation rate [52]. Xylanase can assist in the release of cellulases stuck on the substrate [53] and relieve xylo-oligosaccharide-induced cellulase inhibition [54, 55]. To date, most industrial processes have used GH11 xylanases for biorefining purposes [56]. The XYL10C-ΔN produced in this study has the potential to be applied for biorefining. It not only shows a synergistic activity with cellulase to degrade corn stover, but also enhances the reducing sugar-producing rate and increases the total amount of reducing sugar. We inferred that XYL10C-ΔN could remove xylan obstacles that impede the processive movement of cellulases, involving cleaving xylan into smaller chain products, which would ultimately promote corn stover degradation [17, 57]. Therefore, XYL10C-ΔN has great potential for application in biomass degradation and biofuels.

## Conclusions

In the present study, we obtained a xylanase variant XYL10C-ΔN with excellent thermostability, high catalytic efficiency, and synergistic action with cellulase in the degradation of straw. A key residue on loop 3, Glu175, was found to interact with Lys135 and Met137 of loop 2, and could account for the improved catalytic efficiency and broadened pH-activity profile. This study not only provides an excellent candidate xylanase for potential industrial use, but also provides the theoretical and practical basis for the modification of GH10 xylanases.

## Methods

### Protein preparation

The gene fragment coding for the truncated XYL10C (GenBank Accession Number: FJ492963) without the N-terminal 66 amino acid residues, designated *xyl10c*-ΔN, was amplified by PCR with a primer set (5′-GAATTCTGGGGTCTTAATAATGCAGCTCGAGCCG-3′ and 5′-GCGGCCGCTCATGGACTTTCCGCCTTATGTTGCAAAGCCTG-3′, containing the *Eco*RI and *Not*I restriction sites). The gene fragment was then cloned into the expression vector pPIC9 at the *Eco*RI and *Not*I restriction sites using the T_4_ ligase. The recombinant plasmid pPIC9-*xyl10c*-ΔN was linearized by *Bgl*II and transformed into *P. pastoris* GS115 competent cells using a Gene Pulser X cell Electroporation System (Bio-Rad). Recombinant expression and fermentation were conducted following the method previously described [30].

The culture supernatants were collected by centrifugation (12,000×*g*, 4 °C, and 10 min), followed by concentration through a Vivaflow ultrafiltration membrane (Vivascience) with a molecular weight cut-off of 10 kDa. The crude enzymes were loaded onto a FPLC HiTrap Q Sepharose XL 5 mL column (GE Healthcare) that was equilibrated with 20 mM McIlvaine buffer (pH 6.5). Enzymes were eluted utilizing a linear gradient of NaCl (1.0 M) in the same buffer at a flow rate of 4.0 mL/min. Fractions exhibiting xylanase activities were collected and subjected to sodium dodecyl sulfate-polyacrylamide gel electrophoresis (SDS-PAGE). The protein concentration was determined using the Bradford method with albumin from bovine serum as a standard. To remove *N*-glycans, approximately 600 mg of purified recombinant XYL10C-ΔN was treated by endo-β-*N*-acetylglucosaminidase *H* (Endo *H*_f_, New England Biolabs) in 50 mM sodium citrate (pH 5.6) at 300 K for 1 day. The reaction mixture was then dialyzed twice against 50 mM sodium citrate (pH 6.0) containing 20 mM MES, followed by elution through a DEAE column in 200 mM NaCl. The eluted XYL10C-ΔN was re-dialyzed in 50 mM sodium citrate (pH 7.5) containing 25 mM Tris–HCl, 150 mM NaCl, and concentrated to 66 mg/mL using Amicon Ultra-15 Centrifugal Filter Units (Millipore). The protein purity was higher than 95% as shown by SDS-PAGE.

### Crystallization and data collection

The initial crystallization screen was performed manually using the 768 different reservoir conditions from Hampton Research kits (Laguna Niguel) and sitting-drop vapor-diffusion method. Briefly, 2 μL of XYL10C-ΔN solution (25 mM Tris–HCl [pH 7.5] and 150 mM NaCl; 66 mg/mL) was mixed with 2 μL of reservoir solution in 24-well Cryschem Plates (Hampton Research), and equilibrated against 300 μL of reservoir solution at 298.15 K. The initial crystals of XYL10C-ΔN were obtained within 5 days using the Index condition No. 83 (0.2 M MgCl_2_·6H_2_O, 0.1 M Bis–Tris [pH 6.5], and 25% [w/v] PEG 3350). The crystallization conditions were then optimized to 0.4 M MgCl_2_·6H_2_O, 0.1 M Bis–Tris (pH 6.5), and 19% (w/v) PEG 3350. At day 5, the crystals reached to a size of approximately 1.0 mm × 0.4 mm × 0.3 mm. The crystals that diffracted to 1.5 Å resolution were cryo-protected with a solution containing 0.5 M MgCl_2_·6H_2_O, 0.15 M Bis–Tris (pH 5.5), 25% (w/v) polyethylene glycol 3350 and 10% (w/v) glycerol. Complete X-ray intensity data were collected from a single crystal at beam line BL15A1 of the National Synchrotron Radiation Research Center of China. A 0.5° oscillation angle was used, the exposure time was 3 s, and the distance between crystal and detector was 200 mm. The diffraction images were processed using the program *HKL*-2000 [58]. The XYL10C-ΔN structure was solved using the molecular replacement (MR) method of *Phaser* program [59] in the CCP4 suite with the endo-1,4-β-xylanase XylE from *Phialocephala scopiformis* (53% sequence identity) as a search model (PDB code: 4F8X). Initial structure refinement using *REFMAC5* resulted in a model with the *R*_work_ and *R*_free_ values of 0.125 and 0.175, respectively.

### Site-saturation mutagenesis and enzyme production

Based on the structure analysis and sequence evolution analysis of 50 GH10 xylanases (Table 2), Glu175 of XYL10C-ΔN was found to be special and probably play a key role in the efficient catalysis. Overlap PCR was then performed to substitute Glu175 of XYL10C-ΔN with other amino acid residues. The specific primers are shown in Additional file 1: Table S6. Construction of recombinant plasmids and heterologous expression and purification followed the same procedures as described above.

### Biochemical characterization of the XYL10C-ΔN and its mutants

Beechwood xylan from Sigma was used as the substrate. The pH-activity profiles of the wild-type and variant enzymes were determined by measuring the xylanase activity after incubation at 80°C in McIlvaine buffer (pH 2.5–8.5) and glycine–NaOH (9.0–11.0) containing 1.0% beechwood xylan for 10 min. To determine the optimal temperature, the enzyme activities were measured at temperatures ranging from 30 to 95 °C in McIlvaine buffer of each optimal pH for 10 min. The amounts of reducing sugar released were determined using the 3,5-dinitrosalicylic acid (DNS) method [60]. Thermal stability was determined by measuring the residual activities under standard conditions (pH 4.0, 80 °C, and 10 min) after incubation of the enzymes at 80 and 90 °C for 0, 2, 5, 10, 20, 30, and 60 min. The melting temperature (*T*_m_, thermodynamic stabilities) of XYL10C and XYL10C-ΔN were determined on a MicroCal™ VP-Capillary DSC (GE Healthcare). Each protein sample, 200 μg, were dissolved in 0.5 mL of 20-mM McIlvaine buffer (pH 6.5). The degassed protein samples and controls were treated with a heating rate of 120 °C/h over the temperature range of 20–110 °C. Each experiment was repeated at least three times.

### Analysis of the hydrolysis products

The hydrolysis products of xylo-oligosaccharides and beechwood xylan by XYL10C and XYL10C-ΔN were determined using the high-performance anion-exchange chromatography (HPAEC; Thermo Fisher Scientific, Sunnyvale, CA) equipped with a CarboPac PA200 guard column (3 mm × 250 mm). Each enzyme (0.2 U) was added into the 0.4 mL 50 mM Na_2_HPO_4_-citric acid (pH 4.5) containing 2 μg/mL of xylobiose to xylohexaose or 1% (w/v) beechwood xylan, and incubated at 70 °C for 16 h. The hydrolysis products were resolved in a mobile phase of 100 mM NaOH using xylose to xylohexaose as standards.

### Determination of the kinetic values

Beechwood xylan of 1–10 mg/mL in 0.1 M Na_2_HPO_4_-citric acid buffer was used as the substrate for determination of the *K*_m_, *V*_max_ and *k*_cat_ values. The enzymatic activities of purified recombinant XYL10C-ΔN and its mutants were assayed under standard conditions (80 °C and pH 4.0) for 5 min. The data were plotted and calculated according to the Lineweaver–Burk method. The catalytic efficiency (*k*_cat_/*K*_m_) of each enzyme was calculated for comparison. Significant differences of the *K*_m_ and *k*_cat_/*K*_m_ values were tested using the ANOVA of SPSS 15.0 at the *P* < 0.05 level.

### Verification of the functional roles of Glu175 in other xylanases

To verify the functional roles of Glu175 in other GH10 xylanases, the corresponding residue of XylE from *P. scopiformis* and XynE2 from *Anoxybacillus* sp. E2 were replaced with glutamic acid or other amino acid residues. The gene fragments of *xylE* and *xynE2* (GenBank Accession Number: FJ860894 and GQ240232) without the signal peptide-coding sequence were synthesized by Biomed (Beijing, China). The gene fragments harboring mutations were obtained by overlap PCR with specific primers (Additional file 1: Table S6). For the XylE and its mutants, construction of recombinant vector, expression in *P. pastoris*, purification and characterization followed the same procedures as described above. The standard assay conditions for XylE and its mutants were 70 °C and pH 5.0. For XynE2 and its mutant, the gene fragment and vector pET-22b(+) (Novagen) were digested by *Nco*I and *Hind*Ш, ligated by T_4_ ligase, and transformed into *E. coli* BL21 (DE3) competent cells for expression. The standard assay conditions for XynE2 and its mutant were 65 °C and pH 7.5.

### Enzymatic hydrolysis of corn stover

Corn stover was treated with 15% (w/w) ammonia solution for 24 h as previously reported [61]. Pretreated corn stover was solved in 50 mM sodium citrate (pH 5.0) at the concentration of 2% (*w/v*), followed by the addition of 100 U of *T. reesei* cellulase or 30 U of XYL10C or XYL10C-ΔN and 70 U of *T. reesei* cellulase. The reactions were incubated at 50 °C with an agitation rate of 165 rpm for 24 h. The amounts of reducing sugars released were determined at 1, 2, 4, 8, 12, 18, and 24 h using the DNS method. The experiment was performed in triplicate.

## Additional file

**Additional file 1: Table S1.** X-Ray data collection and structure refinement statistics. **Table S2.** Temperature and pH optima of XYL10C-ΔN and its mutants with beechwood xylan as the substrate. **Table S3.** Specific activity of XYL10C-ΔN and its mutants with beechwood xylan as the substrate under standard conditions (80 °C and pH 4.0). **Table S4.** Temperature and pH optima of XylE and its mutants with beechwood xylan as the substrate. **Table S5.** Specific activity of XylE and its mutants with beechwood xylan as the substrate under standard conditions (70 °C and pH 5.0). **Table S6.** Primers used in this study. **Figure S1.** Multiple sequence alignments of XYL10C-ΔN and other GH10 xylanases using the ClustalW and ESPript. Strictly conserved residues are marked in red. Similar residues are shown in black bold characters and marked in yellow. The catalytic residues are indicated by red diamonds. Some important amino acids are indicated by red triangles. α-Helices and β-strands are represented by black coils and arrows, respectively. **Figure S2.** SDS-PAGE analysis of the purified recombinant XYL10C, XYL10C-ΔN, XylE, XynE2, and their mutants. Lanes: M, the standard protein molecular weight markers; 1, 3, 10, and 45, the crude enzyme of wild-type XYL10C, XYL10C-ΔN, XylE and XynE2; 2, 4, and 11, the deglycosylated XYL10C, XYL10C-ΔN, and XylE; 5-9 and 13-26, the saturated mutants of XYL10C-ΔN; 12 and 27-44, the saturated mutants of XylE; and 46, the mutant XynE2-Q85E. **Figure S3.** Comparison of thermostability of XYL10C and XYL10C-ΔN. A, the kinetic stability assayed at 80 °C and 90 °C; B, the thermodynamic stability (*T*_m_ values). **Figure S4.** pH stability of XYL10C and XYL10C-ΔN. **Figure S5.** HPLC analysis of the hydrolysis products by XYL10C (*A*) and XYL10C-ΔN (*B*). X1, xylose; X2, xylobiose; X3, xylotriose; X4, xylotetraose; X5, xylopentaose; X6, xylohexaose; beechwood, beechwood xylan. **Figure S6.** The X-ray diffraction pattern of the XYL10C-ΔN crystal. The resolution edges are shown by different concentric circles. **Figure S7.** The dimeric structure (Chains A and B) of the XYL10C-ΔN. *A*, The hydrogen bonds between monomers. The residues involved in forming hydrogen bonds are indicated, and the red dotted line represents hydrogen bond. *B*, The van der Waals force network between monomers. The residues in blue are from chain A, and those red are from chain B. The residues involved are represented by yellow dashed lines. **Figure S8.** The XYL10C-ΔN docked with xyloheptaose. The enzyme and substrate interactions are indicated by yellow dotted lines. **Figure S9.** The active-site architecture of mutant XylE-Q116E.

## Abbreviations

SDS-PAGE: sodium dodecyl sulfate-polyacrylamide gel electrophoresis
DNS: 3,5-dinitrosalicylic acid
RT-PCR: reverse-transcription PCR
ORF: open reading frame
MD: minimal dextrose medium
BMGY: buffered glycerol-complex medium
BMMY: buffered methanol-complex medium
BSA: albumin from bovine serum
Endo *H*_f_: endo-β-*N*-acetylglucosaminidase *H*
MR: molecular replacement
