# Supplementary material for: Insight into the functional roles of Glu175 in the hyperthermostable xylanase XYL10C-ΔN through structural analysis and site-saturation mutagenesis
Source: Biotechnol Biofuels. 2018 Jun 8;11:159. doi: 10.1186/s13068-018-1150-8 (PMC5992652; doi:10.1186/s13068-018-1150-8)
Supplement: Supplementary file 1 — Additional file 1: Table S1. X-Ray data collection and structure refinement statistics. Table S2. Temperature and pH optima of XYL10C-ΔN and its mutants with beechwood xylan as the substrate. Table S3. Specific activity of XYL10C-ΔN and its mutants with beechwood xylan as the substrate under standard conditions (80 °C and pH 4.0). Table S4. Temperature and pH optima of XylE and its mutants with beechwood xylan as the substrate. Table S5. Specific activity of XylE and its mutants with beechwood xylan as the substrate under standard conditions (70 °C and pH 5.0). Table S6. Primers used in this study. Figure S1. Multiple sequence alignments of XYL10C-ΔN and other GH10 xylanases using the ClustalW and ESPript. Strictly conserved residues are marked in red. Similar residues are shown in black bold characters and marked in yellow. The catalytic residues are indicated by red diamonds. Some important amino acids are indicated by red triangles. α-Helices and β-strands are represented by black coils and arrows, respectively. Figure S2. SDS-PAGE analysis of the purified recombinant XYL10C, XYL10C-ΔN, XylE, XynE2, and their mutants. Lanes: M, the standard protein molecular weight markers; 1, 3, 10, and 45, the crude enzyme of wild-type XYL10C, XYL10C-ΔN, XylE and XynE2; 2, 4, and 11, the deglycosylated XYL10C, XYL10C-ΔN, and XylE; 5-9 and 13-26, the saturated mutants of XYL10C-ΔN; 12 and 27-44, the saturated mutants of XylE; and 46, the mutant XynE2-Q85E. Figure S3. Comparison of thermostability of XYL10C and XYL10C-ΔN. A, the kinetic stability assayed at 80 °C and 90 °C; B, the thermodynamic stability (Tm values). Figure S4. pH stability of XYL10C and XYL10C-ΔN. Figure S5. HPLC analysis of the hydrolysis products by XYL10C (A) and XYL10C-ΔN (B). X1, xylose; X2, xylobiose; X3, xylotriose; X4, xylotetraose; X5, xylopentaose; X6, xylohexaose; beechwood, beechwood xylan. Figure S6. The X-ray diffraction pattern of the XYL10C-ΔN crystal. The resolution edges are shown by different [file 13068_2018_1150_MOESM1_ESM.doc]

**Additional file**

**Insight into the Functional Roles of Glu175 in the Hyperthermostable Xylanase XYL10C-ΔN through Structural Analysis and Site-saturation Mutagenesis**

Shuai You,^1^ Chun-Chi Chen,^2,3^ Tao Tu,^1^ Xiao-yu Wang,^1^ Rui Ma,^1^ Hui-yi Cai,^1^ Rey-Ting Guo,^2,3*^ Hui-ying Luo,^1*^ Bin Yao^1*^

*^1^*Key Laboratory for Feed Biotechnology of the Ministry of Agriculture, Feed Research Institute, Chinese Academy of Agricultural Sciences, Beijing 100081, China *^2^*National Engineering Laboratory of Industrial Enzymes, Tianjin Institute of Industrial Biotechnology, Chinese Academy of Sciences, Tianjin 300308, China *^3^*College of Life Sciences, Hubei University, Wuhan 430062, China

*Correspondence to Dr. H. Luo, B. Yao and R.T. Guo

E-mail addresses: luohuiying@caas.cn; binyao@caas.cn; guo_rt@tib.cas.cn.

Tel./fax: +86 10 82106065.

**Additional file 1：**

**Table S1. X-Ray data collection and structure refinement statistics. ^a^**

|  | XYL10C-ΔN | XYL10C-ΔN-xylobiose |
| --- | --- | --- |
| Data co1lection |  |  |
| Space group | C2 | C2 |
| -cell |  |  |
| *a* [Å] | 135.45 | 135.28 |
| *b* [Å] | 83.26 | 83.14 |
| *c* [Å] | 65.27 | 65.39 |
| *α* /*β* /*γ* (°) | 90.00/94.49/90.00 | 90.00/94.25/90.00 |
| Resolution [Å] | 25−1.58 (1.64−1.58) | 25−1.70 (1.76−1.70) |
| Unique reflections | 113828 (11236) | 79183 (7945) |
| Redundancy | 5.3 (5.2) | 4.2 (4.1) |
| Completeness [%] | 98.7 (97.3) | 99.7 (99.9) |
| Average *I*/*σ*(*I*) | 49.7 (5.8) | 45.9 (5.96) |
| R_merge_ [%] ^b^ | 3.8 (28.6) | 4.1 (33.7) |
| Refinement |  |  |
| No. of reflections | 108048 (8188) | 79183 (5844) |
| *R*_work_ (95 % of data) ^b^ | 0.125 (0.181) | 0.171 (0.171) |
| *R*_free_ (5 % of data) ^b^ | 0.175 (0.221) | 0.194 (0.200) |
| r.m.s.d. bonds [Å] | 0.012 | 0.762 |
| r.m.s.d. angles [º] | 1.498 | 1.070 |
| Dihedral angles |  |  |
| Most favored [%] | 97.9 | 98.5 |
| Allowed [%] | 2.0 | 1.51 |
| Disallowed [%] | 0.2 | 0.0 |
| No. of non-H atoms / average B [Å^2^] |  |  |
| Protein | 5338/26.01 | 5328/32.74 |
| Water | 788/37.85 | 628/42.54 |
| NAG | 28/32.41 | 28/41.26 |
| CBI |  | 23/46.60 |

^a^ Values in parentheses are for the highest resolution shell.

^b^R_merge_ = ∑hkl∑i|Ii(hkl)-<I(hkl)>| / ∑hkl∑iIi(hkl).

**Table S2. Temperature and pH optima of XYL10C-ΔN and its mutants with beechwood xylan as the substrate.**

| Enzyme | Optimum temperature  (°C) | Optimum pH | Enzyme | Optimum temperature  (°C) | Optimum pH |
| --- | --- | --- | --- | --- | --- |
| XYL10C-ΔN | 80 | 4.0 | E175C | 80 | 4.5 |
| E175G | 85 | 4.5 | E175D | 80 | 4.5 |
| E175P | 85 | 4.5 | E175F | 95 | 4.5 |
| E175V | 80 | 4.5 | E175L | 85 | 4.5 |
| E175Q | 85 | 5.0 | E175R | 85 | 4.5 |
| E175S | 90 | 4.5 | E175T | 95 | 4.5 |
| E175H | 75 | 5.0 | E175W | 85 | 4.5 |
| E175M | 85 | 4.0 | E175Y | 80 | 4.5 |
| E175A | 80 | 3.5 | E175K | 80 | 4.0 |
| E175I | 90 | 5.0 | E175N | 80 | 4.0 |

**Table S3. Specific activity of XYL10C-ΔN and its mutants with beechwood xylan as the substrate under standard conditions (80 °C and pH 4.0).**

| Enzyme | Specific activity  (U/mg) | Enzyme | Specific activity  (U/mg) |
| --- | --- | --- | --- |
| XYL10C-ΔN | 8700 ± 403 | E175C | 1800 ± 71 |
| E175G | 3000 ± 141 | E175D | 2400 ± 101 |
| E175P | 990 ± 44 | E175F | 2600 ± 108 |
| E175V | 300 ± 14 | E175L | 260 ± 11 |
| E175Q | 3600 ± 167 | E175R | 2800 ± 110 |
| E175S | 4800 ± 231 | E175T | 2500 ± 103 |
| E175H | 990 ± 41 | E175W | 1000 ± 21 |
| E175M | 2700 ± 127 | E175Y | 950 ± 43 |
| E175A | 1200 ± 51 | E175K | 1700 ± 77 |
| E175I | 460 ± 20 | E175N | 3000 ± 132 |

^a^ Values represent means ± SD (n = 3) relative to the untreated control samples.

**Table S4. Temperature and pH optima of XylE and its mutants with beechwood xylan as the substrate.**

| Enzyme | Optimum temperature (°C) | Optimum pH | Enzyme | Optimum temperature (°C) | Optimum pH |
| --- | --- | --- | --- | --- | --- |
| XylE | 70 | 5.0 | Q116C | 65 | 5.0 |
| Q116E | 70 | 5.5 | Q116K | 65 | 5.0 |
| Q116A | 70 | 5.0 | Q116R | 65 | 5.0 |
| Q116H | 70 | 5.0 | Q116T | 65 | 5.0 |
| Q116S | 70 | 5.0 | Q116W | 65 | 5.0 |
| Q116M | 70 | 5.0 | Q116D | 65 | 4.5 |
| Q116F | 65 | 5.5 | Q116L | 65 | 4.5 |
| Q116I | 65 | 5.5 | Q116P | 60 | 5.5 |
| Q116N | 65 | 5.5 | Q116V | 60 | 5.5 |
| Q116Y | 65 | 5.5 | Q116G | ND | ND |

ND, no xylanase activity detected.

**Table S5. Specific activity of XylE and its mutants with beechwood xylan as the substrate under standard conditions (70 °C and pH 5.0).**

| Enzyme | Specific activity  (U/mg) | Enzyme | Specific activity  (U/mg) |
| --- | --- | --- | --- |
| XylE | 620 ± 28 | Q116C | 570 ± 24 |
| Q116E | 2300 ± 108 | Q116K | 790 ± 34 |
| Q116A | 1700 ± 88 | Q116R | 710 ± 32 |
| Q116H | 740 ± 38 | Q116T | 840 ± 38 |
| Q116S | 1500 ± 77 | Q116W | 600 ± 27 |
| Q116M | 2200 ± 102 | Q116D | 480 ± 22 |
| Q116F | 740 ± 35 | Q116L | 630 ± 29 |
| Q116I | 600 ± 31 | Q116P | 510 ± 21 |
| Q116N | 350 ± 17 | Q116V | 300 ± 13 |
| Q116Y | 750 ± 28 |  |  |

^a^ Values represent means ± SD (n = 3) relative to the untreated control samples.

**Table S6.** Primers used in this study.

| Primer name | Primer sequence (5′→3′) ^a^ |
| --- | --- |
| XYL10C-ΔN-PF | GGGgaattctggggtcttaataatgcagctcgagccg |
| XYL10C-ΔN-PR | GGGgcggccgctcatggactttccgccttatgttgcaaagcctg |
| E175 Saturation-F | cttatctggcaatccnnNcttcccacatgggttac |
| E175 Saturation-R | catgtgggaagNnnggattgccagataagattgtg |
| XylE-PF | GGGgaattcgccccacatctgcctggtaataaggacatc |
| XylE-PR | GGGgcggccgcttagcaaacggaacaaggtttaccctcc |
| Q116 Saturation-F | cacaacttggtttgggcttctnnMgtctccgacttc |
| Q116 Saturation-R | ggaagtaacgaagtcggagacNnnagaagcccaaacc |
| XynE2-F | GGGCCATGGATATGATTCCGTCGTTACGTGAAGTATAC |
| XynE2-R | GGGAAGCTTACATGTACAAACTTTCCAAAAAGTTGGC |
| XynE2-Q85E-F | ctcgtatggcataacgagactccgacttggatg |
| XynE2-Q85E-R | gtcggagtctcgttatgccatacgagggtatgg |

^a^ The restriction sites are shown underlined; and the mutant sites are shadowed in grey. ^b^ N = A/T/G/C.

**Additional file 2：**


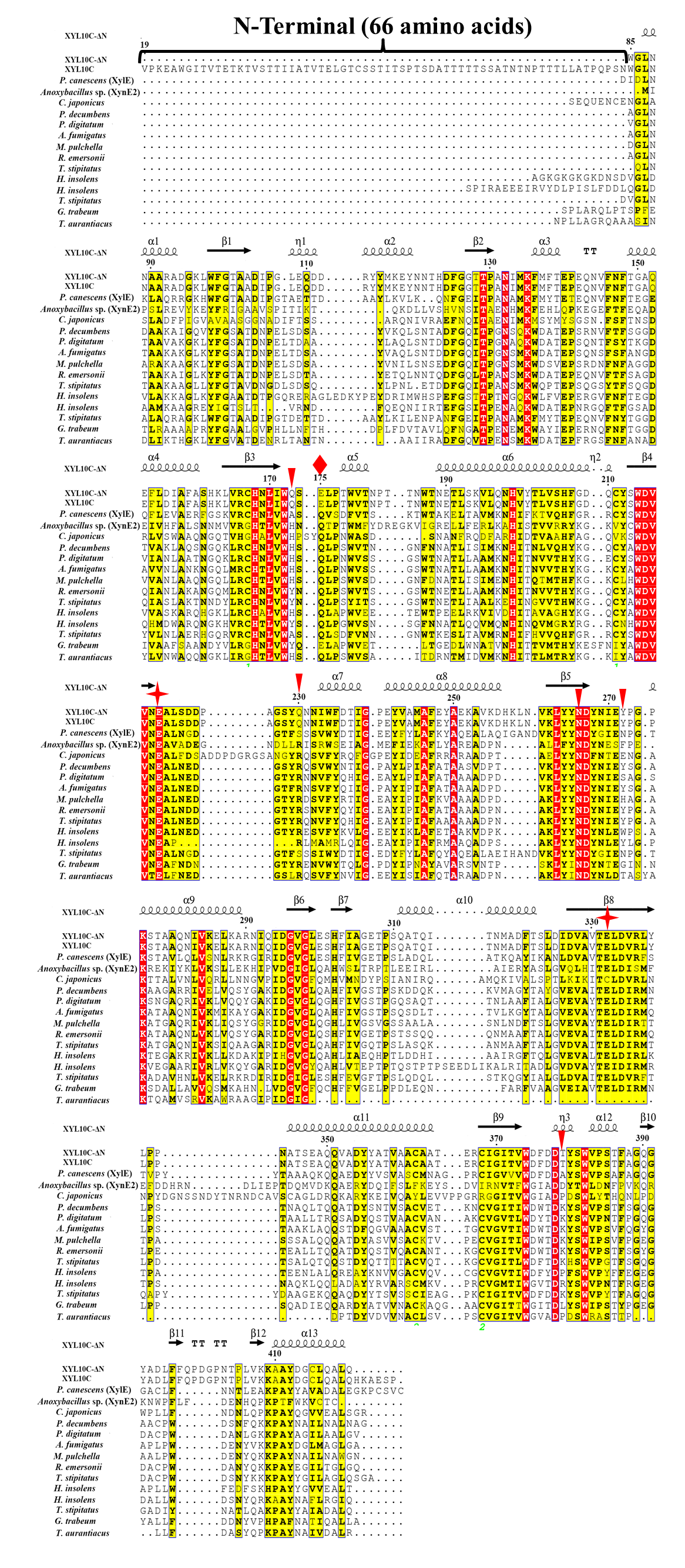


**Fig. S1 Multiple sequence alignments of XYL10C-ΔN and other GH10 xylanases by using the ClustalW and ESPript.** Strictly conserved residues are marked in red. Similar residues are shown in black bold characters and marked in yellow. The catalytic residues are indicated by red diamonds. Some important amino acids are indicated by red triangles. And α-helices and β-strands are represented by black coils and arrows, respectively.


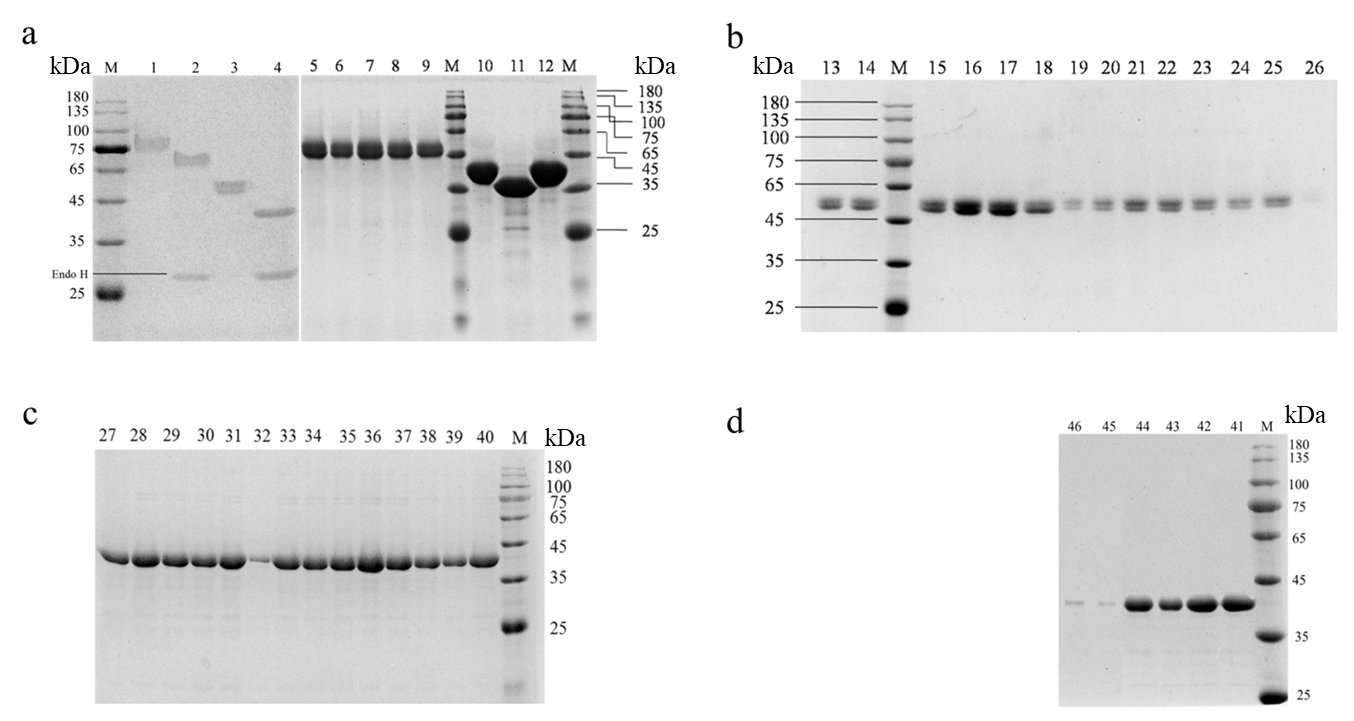


**Fig. S2 SDS-PAGE analysis of the purified recombinant XYL10C, XYL10C-ΔN, XylE, XynE2, and their mutants.** Lanes: M, the standard protein molecular weight markers; 1, 3, 10, and 45, the crude enzymes of wild type XYL10C, XYL10C-ΔN, XylE, and XynE2; 2, 4, and 11, the deglycosylated XYL10C, XYL10C-ΔN, and XylE; 5−9 and 13−26, the saturated mutants of XYL10C-ΔN; 12 and 27−44, the saturated mutants of XylE; and 46, the mutant XynE2-Q85E.

**
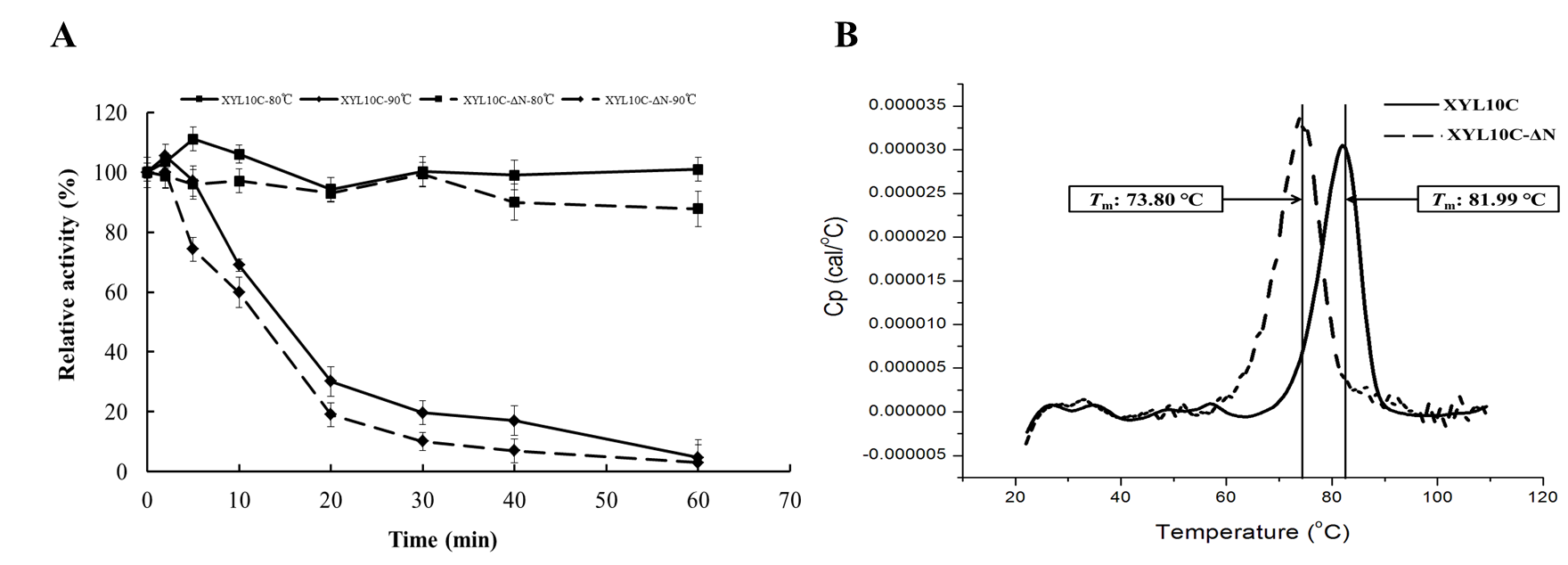
**

**Fig. S3 Comparison of thermalstability of XYL10C and XYL10C-ΔN**. A, the kinetic stability assayed at 80 °C and 90 °C; B, the thermodynamic stability (*T*_m_ values).


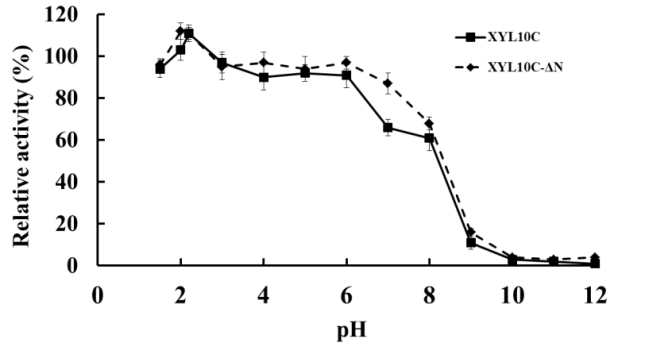


**Fig. S4 pH stability of XYL10C and XYL10C-ΔN.**

**
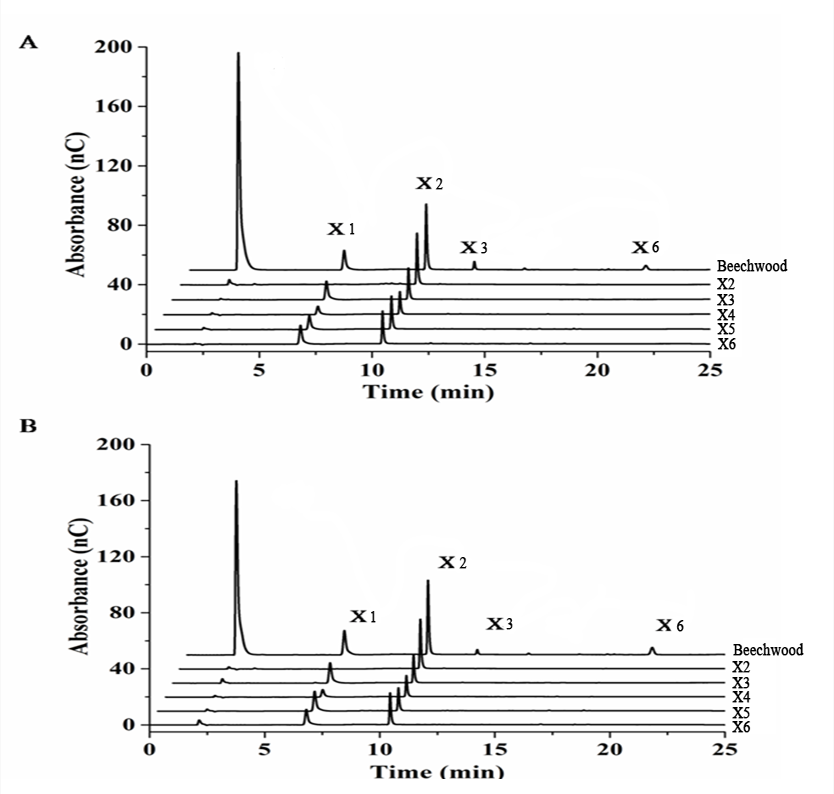
**

**Fig. S5 HPLC analysis of the hydrolysis products by XYL10C (***A***) and XYL10C-ΔN (***B***).** X1, xylose; X2, xylobiose; X3, xylotriose; X4, xylotetraose; X5, xylopentaose; X6, xylohexaose; beechwood, beechwood xylan.


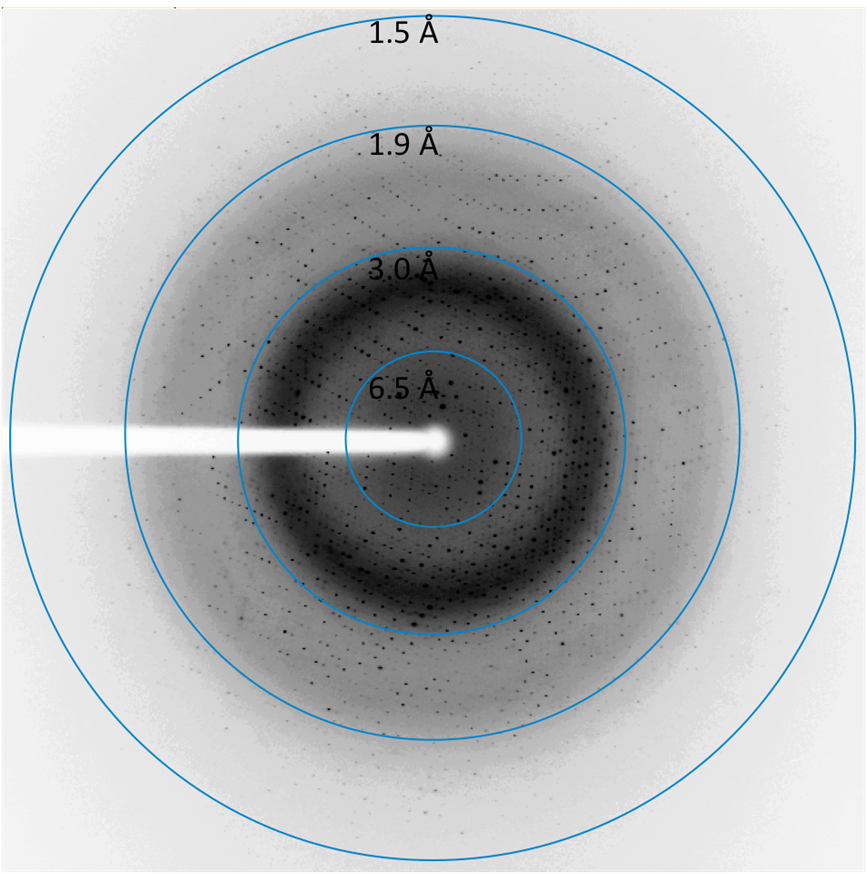


**Fig. S6 The X-ray diffiraction pattern of the XYL10C-ΔN crystal.** The resolution edges are shown by different concentric circles.


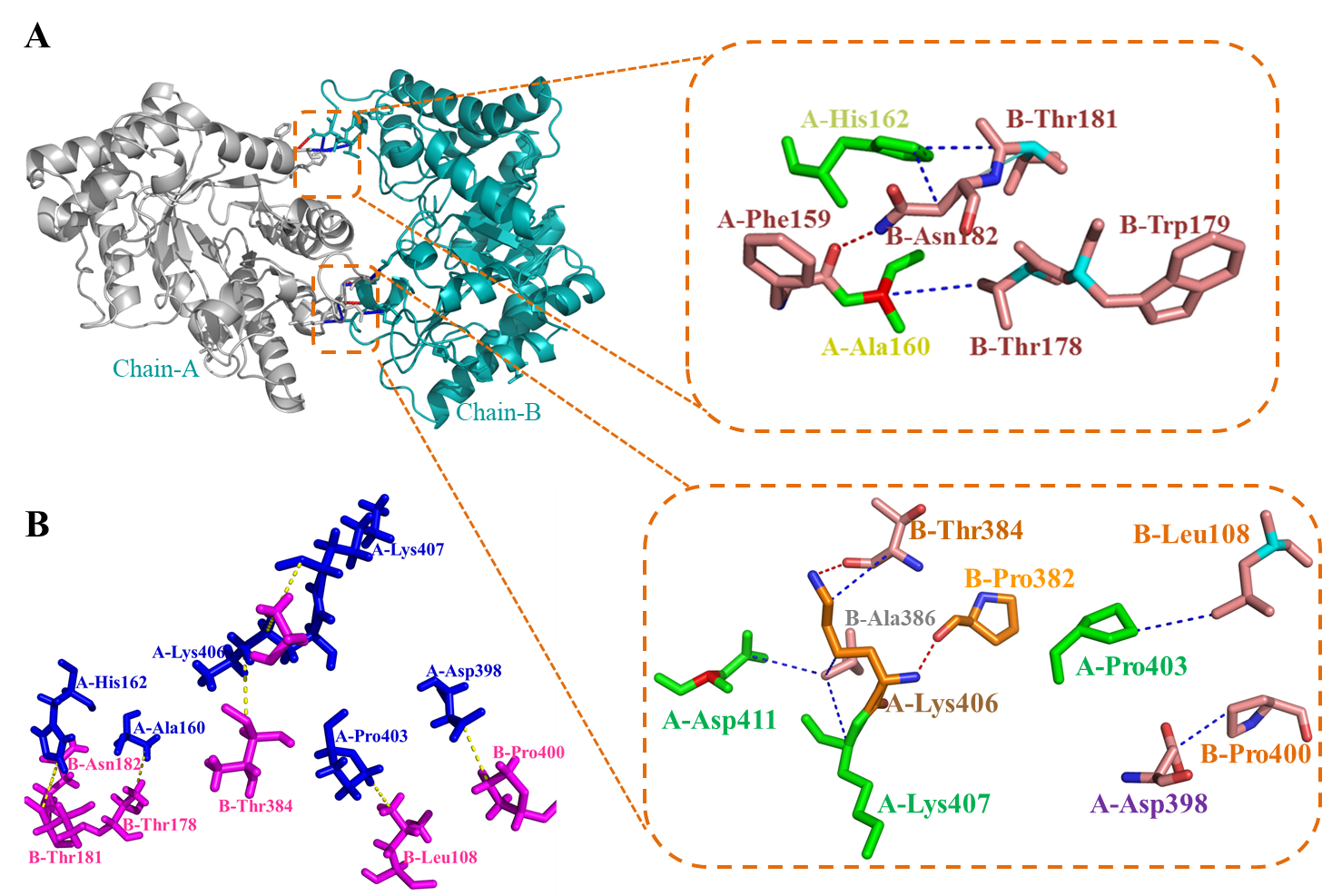


**Fig. S7 The dimeric structure (Chains A and B) of the XYL10C-ΔN.** *A*, The hydrogen bonds between monomers. The residues involved in forming hydrogen bonds are indicated, and the red dotted line represents hydrogen bond. *B*, The van der Waals force network between monomers. The residues in blue are from chain A, and those red are from chain B. The residues involved are represented by yellow dashed lines.

**
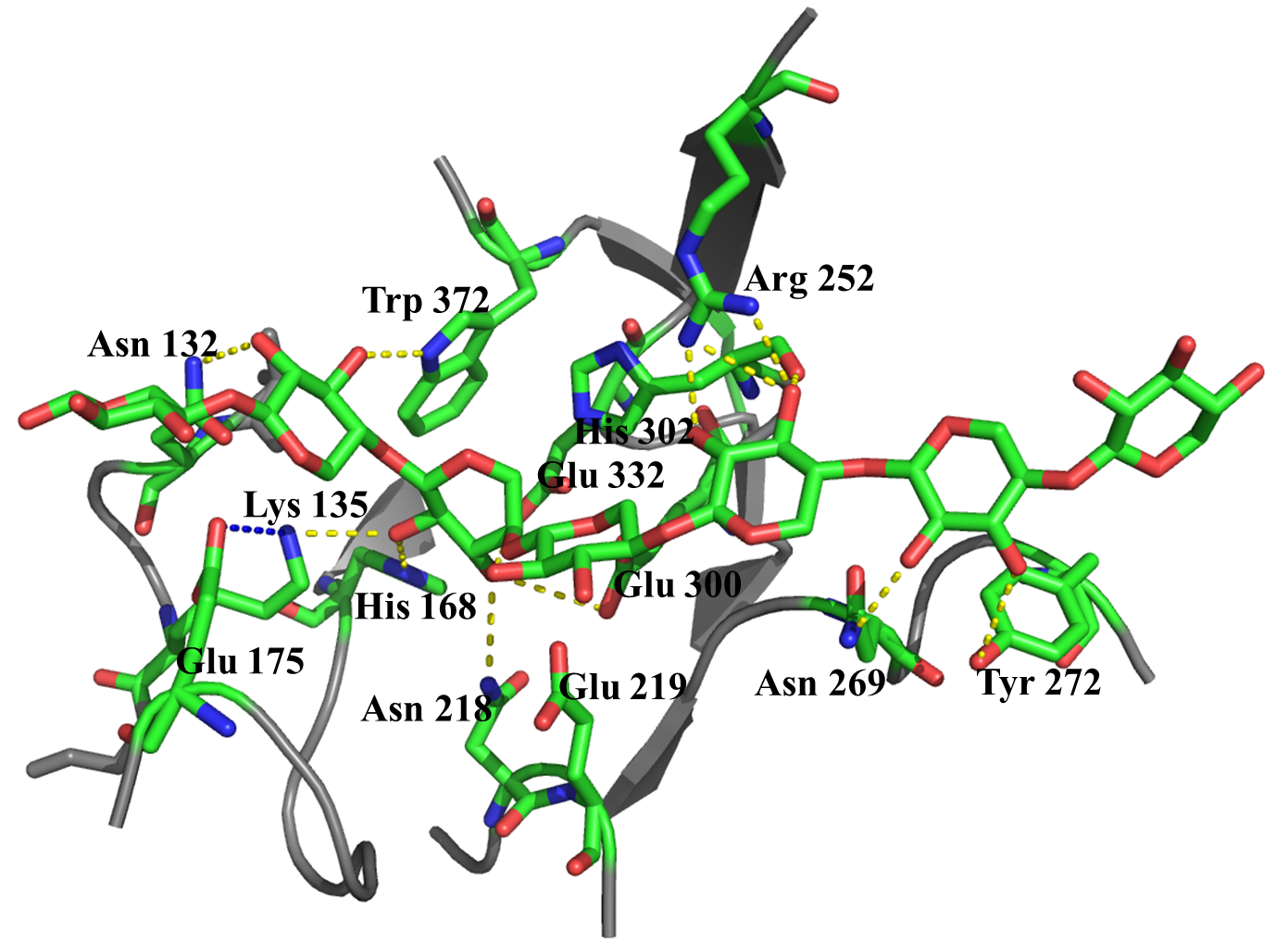
**

**Fig. S8 XYL10C-ΔN is docked with xyloheptaose.** The interaction between substrate and amino acid residues indicated by yellow dotted lines.
